# Outcomes from a mechanistic biomarker multi-arm and randomised study of liposomal MTP-PE (Mifamurtide) in metastatic and/or recurrent osteosarcoma (EuroSarc-Memos trial)

**DOI:** 10.1186/s12885-022-09697-9

**Published:** 2022-06-08

**Authors:** David J. Barnes, Peter Dutton, Øyvind Bruland, Hans Gelderblom, Ade Faleti, Claudia Bühnemann, Annemiek van Maldegem, Hannah Johnson, Lisa Poulton, Sharon Love, Gesa Tiemeier, Els van Beelen, Karin Herbschleb, Caroline Haddon, Lucinda Billingham, Kevin Bradley, Stefano Ferrari, Emanuela Palmerini, Piero Picci, Uta Dirksen, Sandra J. Strauss, Pancras C. W. Hogendoorn, Emmeline Buddingh, Jean-Yves Blay, Anne Marie Cleton-Jansen, Andrew Bassim Hassan

**Affiliations:** 1 Oxford Molecular Pathology Institute, Sir William Dunn School of Pathology, University of Oxford, South Parks Road, and Oxford University Hospital NHS Trust, Oxford, OX1 3RE UK; 2 Nuffield Department of Orthopaedics, Rheumatology and Musculoskeletal Sciences and Centre for Statistics in Medicine (CSM), University of Oxford, Botnar Research Centre, Windmill Road, Oxford, OX3 7LD UK; 3 Institute of Clinical Medicine, Faculty of Medicine, University of Oslo and Department of Oncology-Norwegian Radium Hospital, Oslo University Hospital, Oslo, Norway; 4 Leiden University Medical Center, P.O. Box 9600, Postzone K1-P, 2300RC Leiden, The Netherlands; 5 Department of Oncology Early Phase trials unit and Oncology Clinical Trials Office (OCTO), University of Oxford, Old Road Campus Research Building, Oxford, OX3 7DQ UK; 6 Cancer Research Clinical Trials Unit (Cancer Sciences), Institute of Cancer and Genomic Sciences, Robert Aitken Building, University of Birmingham, Edgbaston, Birmingham, B15 2TT UK; 7 Department of Radiology, Churchill Hospital, Oxford University Hospitals Foundation Trust, Oxford, OX3 7LJ UK; 8 Istituti Ortopedici Rizzoli, Via C. Pupilli 1, 40136 Bologna, Italy; 9 Pediatrics III, West German Cancer Centre Network Essen-Muenster, University Hospital Essen, Hufelanstr 55, Essen, 45147 Germany; 10 Department of Oncology, UCLH NHS Foundation Trust, 250 Euston Road, London, NW1 2PG UK; 11 Universitè Lyon 1 Claude Bernard, Lyon, France

**Keywords:** Osteosarcoma, Muramyl tripeptide, Phase II trial, Bayesian, Sarcoma, Bone neoplasm, Rare cancer

## Abstract

The phase III clinical study of adjuvant liposomal muramyl tripeptide (MTP-PE) in resected high-grade osteosarcoma (OS) documented positive results that have been translated into regulatory approval, supporting initial promise for innate immune therapies in OS. There remains, however, no new approved treatment such as MTP-PE for either metastatic or recurrent OS. Whilst the addition of different agents, including liposomal MTP-PE, to surgery for metastatic or recurrent high-grade osteosarcoma has tried to improve response rates, a mechanistic hiatus exists in terms of a detailed understanding the therapeutic strategies required in advanced disease. Here we report a Bayesian designed multi-arm, multi-centre, open-label phase II study with randomisation in patients with metastatic and/or recurrent OS, designed to investigate how patients with OS might respond to liposomal MTP-PE, either given alone or in combination with ifosfamide. Despite the trial closing because of poor recruitment within the allocated funding period, with no objective responses in eight patients, we report the design and feasibility outcomes for patients registered into the trial. We demonstrate the feasibility of the Bayesian design, European collaboration, tissue collection with genomic analysis and serum cytokine characterisation. Further mechanistic investigation of liposomal MTP-PE alone and in combination with other agents remains warranted in metastatic OS.

**Supplementary Information:**

The online version contains supplementary material available at 10.1186/s12885-022-09697-9.

Osteosarcoma (OS) is the most common high-grade primary tumour arising from bone, where proliferating mesenchymal tumour cells produce osteoid. OS commonly arises in the metaphyses of long bones, and frequently presents with the symptom of pain. RARECARE estimate 0.23–0.5 per 100,000 people in the European Union (EU27) are diagnosed with OS per year [1]. The peak age of OS diagnosis is 15–17 years. Current treatments for osteosarcoma achieve 54 and 71% 5-year event-free survival (EFS) and overall survival for the patients who present with localized disease, respectively [2]. Survival is much lower for metastatic OS, with approximately 28% 5 year EFS for the patients with clinically detectable metastatic disease [2]. Surgical resection of all clinically detectable sites of disease and concomitant first-line systemic chemotherapy with cisplatin, doxorubicin and high-dose methotrexate remains the standard of care [3, 4]. Since the improvement associated with neo-adjuvant and adjuvant chemotherapy to surgery in the early 1980s, the long-term survival of patients with osteosarcoma remains the same despite large scale clinical trials testing additional agents such as interferon and ifosfamide (EURAMOS) [3, 4]. Pulmonary metastases continue to be the major cause of death in patients with osteosarcoma [5–7]. Most recurrences appear in the first two years following diagnosis, with a more dismal outcome if the patient is still on first line chemotherapy. Although patients with metastatic disease can be rendered disease-free by pulmonary metastectomy, the recurrence rate is high at 60–70% within 1 year [5]. Since salvage chemotherapy has had little impact on the disease-free interval in this group of patients, new therapies are required in either potentially resectable or irresectable metastatic OS [8].

Circumstantial evidence suggests that the innate immune system may be important in OS. Deep postoperative bacterial infection appears an independent prognostic factor in OS patients. Patients who have bacterial infection appear to have a 10-year survival of 84.5% compared with 62.2% in patient with no infection [9]. One mechanistic explanation for this observation is that there may be anti-tumour activity of bacterial cell wall muramyl dipeptides (MDP) stimulating innate immune cells. Originally discovered by Coley (Coley’s toxin), MDP can lead to activation of macrophages and monocytes to release local anti-tumour cytokines [10]. For example, MDP is the minimal structural unit of immune potentiating activity from the cell wall of the bacteria, e.g. from Bacille Calmette-Guerin. Activation of the innate immune system in OS using purified bacterial cell wall was confirmed following successful treatment in dogs. In 1989, MacEwen reported that dogs with OS that received muramyl tripeptide ethanolamine (MTP-PE) had a significant prolongation of EFS (*p* < 0.001) compared with dogs that did not receive MTP-PE [11]. MTP-PE is a fully synthetic lipophilic derivative MDP [11]. MTP-PE has similar immune-stimulatory effects as the natural MDP through the pattern recognition receptor pathway, with the additional advantage of a longer half-life in plasma and lower toxicity. The term ‘liposomal MTP-PE’ is used for a specific well-defined liposomal-encapsulated formulation of the active MTP-PE. The encapsulation of MTP-PE into liposomes has shown in vitro to enhance the activation of murine macrophages and human monocytes by 100-fold compared with free MDP, that liposomal MTP-PE is ten-times less toxic than free drug substance [12, 13].

In early phase trials, monocyte/macrophage activation occurred in 24 out of 28 cancer patients following intravenous infusion of liposomal MTP-PE [14–17]. The whole-body distribution of 99mTc-labeled liposomal MTP-PE confirmed that liposome accumulation in lungs of patients was similar to that observed in mice. Uptake in the liver, spleen, lung, nasopharynx and thyroid was observed in four out of four patients six hours after intravenous infusion, and in two patients, liposome accumulation in or around lung metastases was also observed [18]. Toxicity was limited to flu-like symptoms including fever, chills, fatigue, and myalgias. The maximum tolerated dose was 6 mg/m^2^, much higher than the optimal biological dose of 2 mg/m^2^ as assessed by increased monocyte cytotoxicity, serum interleukin 1, interleukin 6, and C-reactive protein levels [17, 19]. Between 1993 to 1997, the Children’s Oncology Group (COG) conducted a prospective, randomized phase III trial (INT-0133) of 600 patients in newly diagnosed OS in patients who were younger than 30 years [20, 21]. The study proposed two questions; the first was whether addition of ifosfamide to doxorubicin, cisplatin and high-dose methotrexate would improve event free survival (EFS). The second question was whether addition of liposomal MTP-PE to chemotherapy would improve EFS. The first conclusion of this study was that the addition of ifosfamide to MAP did not enhance EFS or overall survival. The second conclusion was that addition of mifamurtide to ifosfamide chemotherapy resulted in a statistically significant improvement in overall survival and a trend toward better EFS. The six-year probability of surviving without a relapse was 66% in patients who received the drug compared with 57% in patients who did not receive the drug [20, 21]. Liposomal MTP-PE (Mifamurtide) is now licensed by the European Medicines Agency in combination with adjuvant chemotherapy for the treatment of high-grade resectable non-metastatic OS after macroscopically complete surgical resection in children, adolescents and young adults under 30 years.

The question remains as to how and whether liposomal MTP-PE can also impact the outcomes of either metastatic or advanced OS, with and without concomitant chemotherapy [22, 23]. Further impetus for research into this question followed an evaluation of biopsies from primary and metastatic OS. These revealed that CD14+ macrophages were abundant in OS, with metastatic samples associated with high numbers of CD14+ cells, suggesting that activation of OS specific macrophages may be achievable with activation of innate immunity against OS [24].

Here we report a Bayesian designed multi-arm, multi-centre open-label phase II study in patients with metastatic and/or recurrent OS, designed to investigate if, and why, some patients with OS might respond to liposomal MTP-PE given alone or in combination with ifosfamide. The objective being tested was to identify markers of response to MTP-PE (mifamurtide) by evaluation of radiological response and biological markers of immune response activation in tumour samples, taken before and after six weeks of treatment. The pharmacodynamic readouts were to be compared with radiological (CT) scan response using standard RECIST criteria. Despite the trial closing because of poor recruitment in the funding period, we report the design and feasibility outcomes for the eight OS patients that registered and were either allocated to arm A (MTP-PE/ mifamurtide alone) in resectable disease, or randomised between arm B (Ifosfamide alone) and arm C (Ifosfamide and MTP-PE/ mifamurtide) for unresectable disease.

## Methods

### Study design

**Fig. 1:**
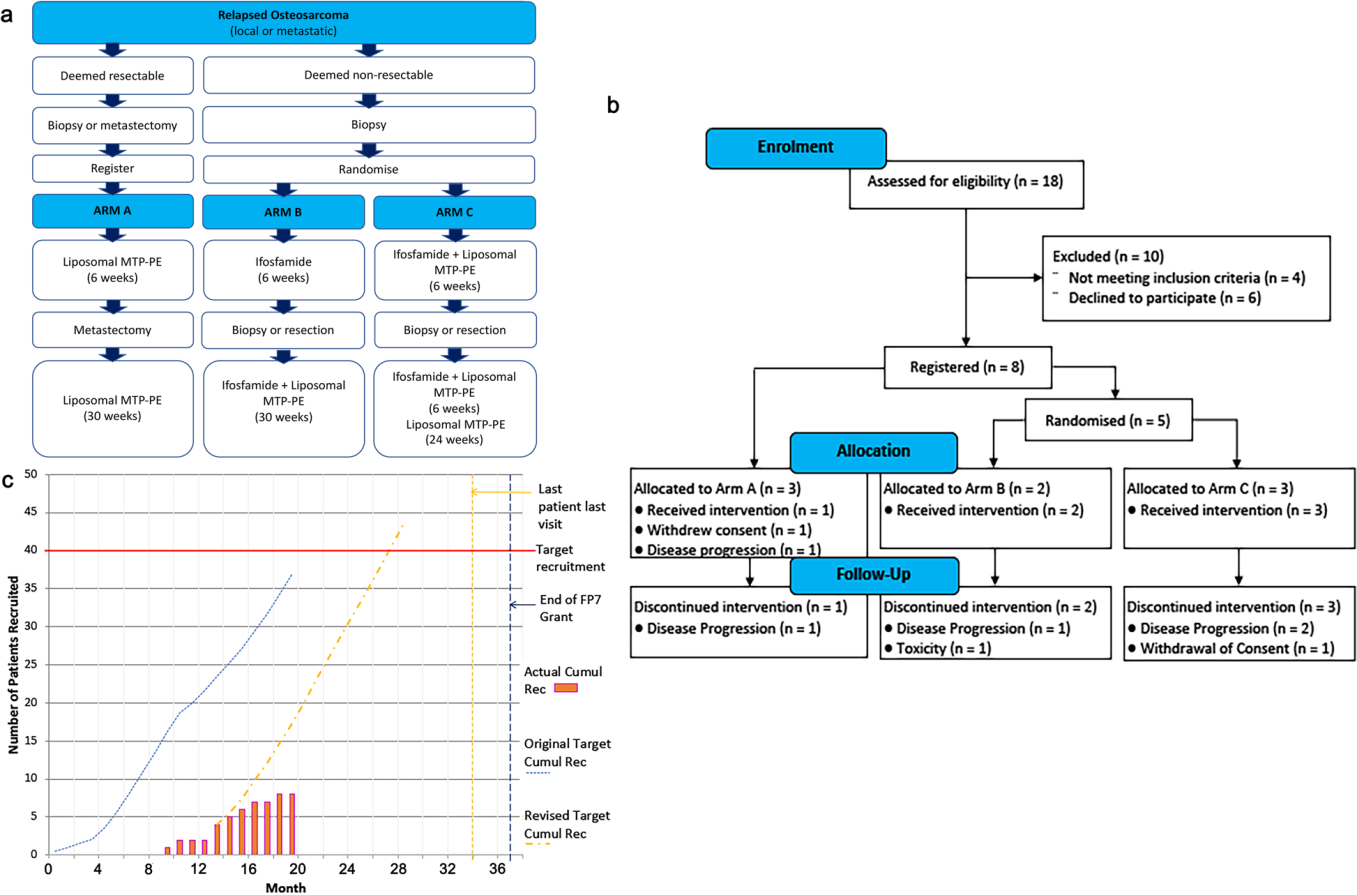
**a**. Flow diagram of the study design with three arms A, B and C. Randomisation is between arm B and C in the deemed un-resectable cohort. **b**. Consort flow diagram of the study participants. A total of 18 patients assessed, 10 were excluded and 8 registered. Of the latter, 5 were randomised. **c**. Planned and actual recruitment during the study period

The trial was an open label Phase II study parallel assignment with no blinding. Depending on their current disease status, patients with biopsy proven high-grade OS were either registered to Arm A (resectable group), to receive liposomal MTP-PE alone; or randomised to Arm B or C (non-resectable group), to receive liposomal MTP-PE or liposomal MTP-PE in combination with ifosfamide, respectively. The primary objectives concerned the radiological and pharmacodynamic response within the first six weeks of treatment. Specifically, Arm A - liposomal MTP-PE alone; Arm B - Ifosfamide alone for 6 weeks then Ifosfamide + liposomal MTP-PE for 6 weeks, then liposomal MTP-PE alone for 30 weeks; Arm C - Ifosfamide + liposomal MTP-PE for 12 weeks then liposomal MTP-PE alone for 24 weeks (Fig. 1a). The intention for all participants was administration of 36 weeks or more of liposomal MTP-PE. Biopsies (or resected tumour samples) obtained before and after 6 weeks of therapy aimed to achieve an interval in order to determine the biological endpoint for liposomal MTP-PE. Scans were planned 6-weekly to assess the radiological response using RECIST version 1.1. The target sample size was 40 patients based on the prior non-informative probabilities and calculation at an interim analysis stage. Randomisation with block sizes of 2 and 4 to arm B or C was performed and communicated by the Sponsor clinical trial office following central registration. The study design was discussed and supported by the OS patient groups in meetings with the Bone Cancer Research Trust in the UK, and the sarcoma patient organisation SPAEN, a partner in the EuroSarc FP7 consortium. Inclusion criteria were adult patients over 16–65 years of age or 18–65 years of age depending on site, histological confirmation of OS, documented measurable, accessible and progressive disease, WHO < 2, cardiac ejection fraction > 45% adequate haematological (Hb > 9 g/dl, ANC > 1.0 × 10^9^, platelets > 80 × 10^9^), renal and liver function and written informed consent.

### Statistical methods

The co-primary endpoints of the study were a biological response and a radiological response. A biological response was based on innate immunity response biomarkers expected to occur in the tumours in response to liposomal MTP-PE. Response was defined as 30% change in gene expression of pattern recognition pathway dependent genes, or similar expression biomarkers, and 30% change in activated macrophage (CD14+) or associated biomarker scores in tumour sections. A radiological response was defined as complete or partial response and assessed using RECIST criteria version 1.1. A patient was defined as a responder if at least one of these two endpoints was met and a non-responder otherwise, on an intention to treat basis. Secondary endpoints included toxicity (CTCAE Criteria (v4.0), systemic levels of activated by liposomal MTP-PE, disease specific survival and progression free survival.

The study comprised of three arms answering two different research question about the efficacy of liposomal MTP-PE in OS patients. Operable patients were allocated to arm A to receive liposomal MTP-PE alone. Inoperable patients were randomised between arms B and C. Patients on these arms both receive Ifosfamide (chemotherapy) and liposomal MTP-PE with arm B starting liposomal MTP-PE 6 weeks later to allow comparison.

#### Design of Arm A

For arm A, a single arm two-stage design was planned. A Bayesian analysis of the accumulated data will be carried out at each analysis to derive the current posterior distribution for the response rate. The posterior predictive probability of a successful trial will be calculated at the interim analysis. Arm A would stop for futility if there is less than a 10% chance of success (90% confidence the trial will fail if continued) whilst it will stop for efficacy if there is greater than a 90% chance of success, otherwise it will continue to recruit. At the final analysis the trial would recommend further research in this patient population if there is a 90% posterior probability that the response rate R is greater than 0.1. The design was based on a non-informative prior distribution for response.

For each cohort we assume the number of patients who respond (R) come from a Binomial distribution R~Binomial(N, p). A non-informative Beta(10^−3^, 10^−3^) prior distribution will be placed on R. Since the Beta distribution is conjugate for binary outcomes the posterior distribution is also a Beta distribution. The chosen design is both a frequentist optimal design and optimal in the Bayesian framework for a 2-stage phase two trial (p0 = 0.1, p1 = 0.3, alpha = 0.1 and power = 0.8 for the frequentist design) requiring a total of 18 patients with the interim analysis after 7 patients.

#### Design of Arms B and C

Eligible patients who were deemed unresectable were randomly assigned (1:1) to arms B and C using block randomisation with block sizes of 2 and 4. The study had an open-label design. Participants, those administering the interventions, and those assessing the outcomes were aware of which treatment had been allocated. Non-resectable patients were randomised between starting liposomal MTP-PE immediately (arm B) and starting liposomal MTP-PE after the second biopsy/resection (week 7) (arm C). This provides a 6-week window to compare patients with and without liposomal MTP-PE. The comparison of arms B and C has not been formally powered and in this sense the investigation is exploratory. A Bayesian analysis of the accumulated data will be carried out to estimate posterior distributions for the response rate on the control arm (B) and the log-odds ratio for the probability of response on arm C compared with arm B. The design is based on non-informative prior distributions. A non-informative Beta(10^−3^, 10^−3^) prior distribution will be used for the response rate on the control arm (B), whilst a Gaussian distribution (N(0,1000)) will be used for the prior distribution of the log-odds ratio. If the posterior probability that the odds ratio is greater than 1 is greater than 80% then we would recommend further research of the liposomal MTP-PE treatment in the unresectable cohort.

### Genomic sequencing and bioinformatics

Germline DNA was extracted from whole blood using a Qiagen QIAamp DNA blood Midi kit in accordance with the manufacturer’s instructions. Samples were shipped to Source BioScience Ltd. (Nottingham, UK) on dry-ice, where quality control analysis was performed using high-sensitivity broad range Qubit assays, with Nanodrop determination of A260/A280 and A260/A230 ratios. Library preparation was with an Agilent SureSelect Human V6 kit, and whole exome sequencing performed on an Illumina HiSeq 4000 (2x150bp per lane). A minimum of three, maximum of four core-biopsies were taken from the patient at each of the 2 time points for pharmacodynamic assays (two cores in neutral buffered formalin, the remainder fresh frozen in RNAlater®). Whole RNA was extracted from biopsies using a Qiagen RNeasy Fibrous Tissue Kit in accordance with the manufacturer’s instructions. Samples were shipped to Source BioScience Ltd. (Nottingham, UK) on dry-ice where quality control analysis was done using an RNA 6000 assay on an Agilent BioAnalyzer 2100.

Three main analysis pipelines were used: transcript abundance (expression) was determined from RNA-Seq data using the Tuxedo pipeline [25], fusion genes were detected using FusionCatcher [26] and variants (single nucleotide variants (SNV) and indels) were detected in RNA-Seq data and whole exome data using the Genome Analysis Toolkit [27]. For analysis of transcript abundance from RNA-Seq data, analyses were carried out using the software tools in the Tuxedo pipeline. Paired-end reads were acquired on a HiSeq 2500 (Illumina) and the Fastq files were aligned to the human genome (hg19/b37) with the TopHat-Bowtie2 aligner, versions 2.0.13 and 2.2.5, respectively. Transcript assembly was done using Cufflinks 2.2.1 and the expression of transcripts was quantified with Cuffdiff and CummeRbund, version 2.2.1, as fragments per kilobase per million mapped reads (FPKM). Statistically significant changes in gene expression were identified using CummeRbund. Gene Set Enrichment Analysis was done using GSEA 3.0 [28]. Putative fusion genes were identified from RNA-Seq data using FusionCatcher, version 0.99.4c, (Kallioniemi Group, Institute for Molecular Medicine, University of Helsinki, Finland). Supporting reads for fusions were inspected and fusions annotated as read-through transcription or involving known fusions between pseudogenes were discarded. Annotation of known oncogenic fusion genes was added by FusionCatcher using data from the Mitelman database (http://cgap.nci.nih.gov/Chromosomes/Mitelman). For whole-exome sequencing of germline (blood) DNA, reads from Fastq files were mapped to the reference human genome (hg19/b37) with the Burrows-Wheeler Aligner (BWA) package, version 0.7.12. Local realignment of the mapped reads around potential insertion/deletion (indel) sites was carried out with the Genome Analysis Tool Kit, version 4.0 (GATK, Broad Institute). Duplicate reads were marked using Picard, version 2.3.0 (Broad Institute). Base quality scores were recalibrated using GATK’s Base recalibration tool and variants (SNVs and indels) were called using the GATK Haplotype Caller. Deleterious SNVs were identified using the Variant Effect Predictor (VEP), release 77 (Ensembl), which uses three algorithms (Sift, Condel and PolyPhen) to predict the functional consequences of mutation. VEP was also used to annotate SNVs for inclusion in dbSNP and COSMIC. Variant detection from RNA-Seq data used a similar pipeline, the main difference being the use of the splicing-aware aligner STAR for the initial mapping of reads [29]. Data from the trial is currently stored on a server operated by the University of Oxford Computational Biology Research Group, with back-ups on external hard drives.

### Cytokine and macrophage activation assays

Bio-Plex multiplex human cytokine 27-plex assay (Bio-Rad Laboraties, Veenendaal, the Netherlands) was utilised for the detection of 27 cytokines and chemokines as per manufacturer’s instructions. 100 μl plasma samples were analysed in duplicate using a Bio-Plex Array Reader with Bio-Plex software.

### Ethical review statement

MEMOS trial (NCT02441309 12/05/2015, ISRCTN46249783, EudraCT 2012–000615-84, EuroSarc-MEMOS) was a Phase II trial containing a randomised comparison sponsored by the University of Oxford and approved by UK national research ethics committee (14/SC/0255) and ratified by ethical review boards of the participant sites across Europe. The trial was co-ordinated by the Oxford Oncology Clinical Trials Office (OCTO). All patients entering the study required written informed consent. The trial had two ethically approved substantial amendments, the first included inclusion of additional new sites across the UK and Europe (22/05/2015) and the second (16/12/2015) included use of alternative GFR and renal function calculations and cardiological screening techniques used by different sites.

## Results

### Study participants

**Fig. 2:**
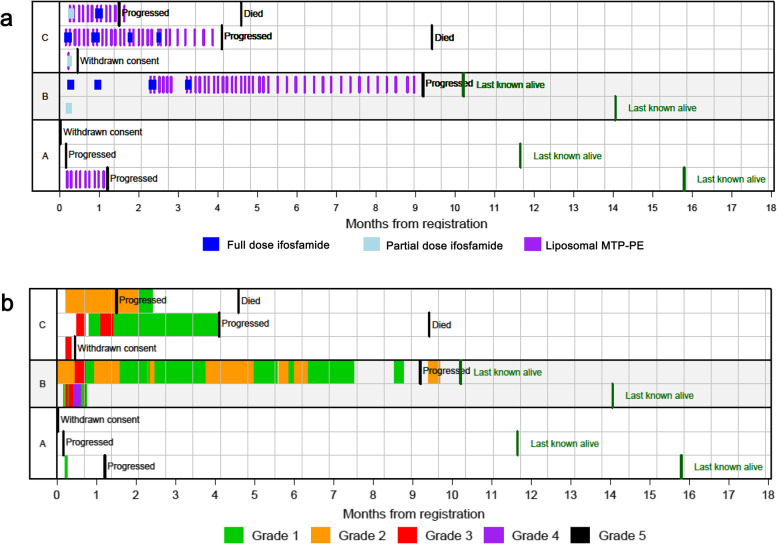
**a**. Summary of treatments administered and major patient events for each patient in all arms. **b**. Summary of the timing and of worst adverse events for each patient in all arms

The trial setting was within bone sarcoma tertiary specialist clinical treatment centres across the UK and Europe, using teams with diagnostic, age appropriate and chemotherapy expertise in osteosarcoma. Fig. 1b displays the consort flow diagram of the study enrolment and Fig. 1c the planned and actual recruitment trajectories. Overall, 18 patients were assessed for eligibility, 4 patients did not meet eligibility criteria, 6 patients declined to participate and 8 registered in the study following written informed consent. Thus, a total of 14 patients met the eligiblity criteria and had resectable/ accessible lesions for tissue and had measurable disease. Of the 4 patients who did not meet eligibility, 1 was because of lack of evidence of progressive disease, 2 had a WHO performance status < 2 and 1 had a low platelet count. Of the 6 patients who declined to participate, all decided that the major reason was because of the travel required to attend the trial site, and the associated number of attendances required over a maximum of 42 weeks. Of the 8 patients that registered, 1 patient in Arm A later withdrew consent and did not start treatment, 1 patient in Arm A developed progressive disease and did not start treatment, and 5 received the allocated intervention; to Arm A 1 out of 3 registered, to Arm B 2 out of 2 registered and to Arm C 3 out 3 registered. Subsequently, 1 further patient in Arm C withdrew consent following toxicity. Table 1 outlines the baseline characteristics of the 8 patients registered. Most patients were male. One patient in Arm A, one in Arm B and three in Arm C received liposomal MTP-PE (Fig. 2a). Patients were recruited from the Churchill hospital Oxford (*n* = 3), Norwegian Radium hospital, Norway (*n* = 2), Leiden University Medical Center, Netherlands (*n* = 1), Instituti Ortopedici Rizzoli (n = 1) and University College hospital, London (n = 1). The challenges that affected recruitment were administrative delays to opening the first site of the study in the UK and subsequent opening of sites in other countries. Due to limitations of public funding for this investigator study and associated cost issues, a single site could only be selected in each country. This led to challenges for the referral pathways, the travel for patients and families undergoing frequent treatment infusions, so leading to the logistic reason for declining to participate and the early withdrawal from the study. Despite additional UK sites aiming to enrol patients, the study closed on advice from the sponsor following poor recruitment over the available funding period.

**Table 1:** Baseline characteristics of the patients recruited

Characteristic		Overall (*n* = 8)
Age (years, Median (Q1,Q3))		24.5 (20.2,34.5)
Gender	Male	7 (87.5%)
	Female	1 (12.5%)
Performance Status	0	6 (75.0%)
	1	2 (25.0%)
Histology/Cytological type	Chondroblastic OS 9181/3	1 (12.5%)
	Osteoblastic OS 9180/3	2 (25.0%)
	Osteosarcoma NOS 9180/3	5 (62.5%)
Primary Site	axial	3 (37.5%)
	limb	5 (62.5%)
Disease stage at screening	metastatic	8 (100.0%)
Tumour size at baseline (sum of longest diameters) (mm)		82.0 (51.0,92.0)
Prior radiotherapy	yes	2 (25.0%)
	no	6 (75.0%)
Prior chemotherapy	yes	8 (100.0%)
	no	0 (0.0%)
Prior surgery	yes	8 (100.0%)
	no	0 (0.0%)

### Primary endpoints

Patients received prescribed treatment as indicated in Fig. 2a. Radiological response at six weeks was one of the two primary study endpoints. There were no complete or partial responders out of the 5 patients that underwent a scan at this time point, with 2 and 3 patients with stable disease and progressive disease, respectively (Table 2). Table 3 and Fig. 2 summarises the time of event data for progression free and overall survival. Supplementary Table 1 provides the CONSORT abstract summary including outcomes.

**Table 2:** Primary response analysis

Trial Number	Scan 1 (week 6)	Scan 2	Scan 3	Scan 4	Scan 5
A1	PD				
A2	PD				
B2	SD	SD	SD	SD	PD
C2	SD	SD	PD		
C3	PD				

*PD* progressive disease

*SD* stable disease

**Table 3:** Time to event (months) progression free and disease specific survival

Patient Number	Treatment Stopped	PFS	OS	Cause of death	Last known alive
A1	1.2	1.2			15.8
A2	Did not start	0.2			11.7
A3	Did not start^a^				0.0^1^
B1	0.8				14.1
B2	9.4	9.2			10.2
C1	0.5				0.5
C2	4.2	4.1	9.4	Disease related	
C3	1.7	1.5	4.6	Disease related	

^a^Withdrew after enrolling into the study

**Fig. 3:**
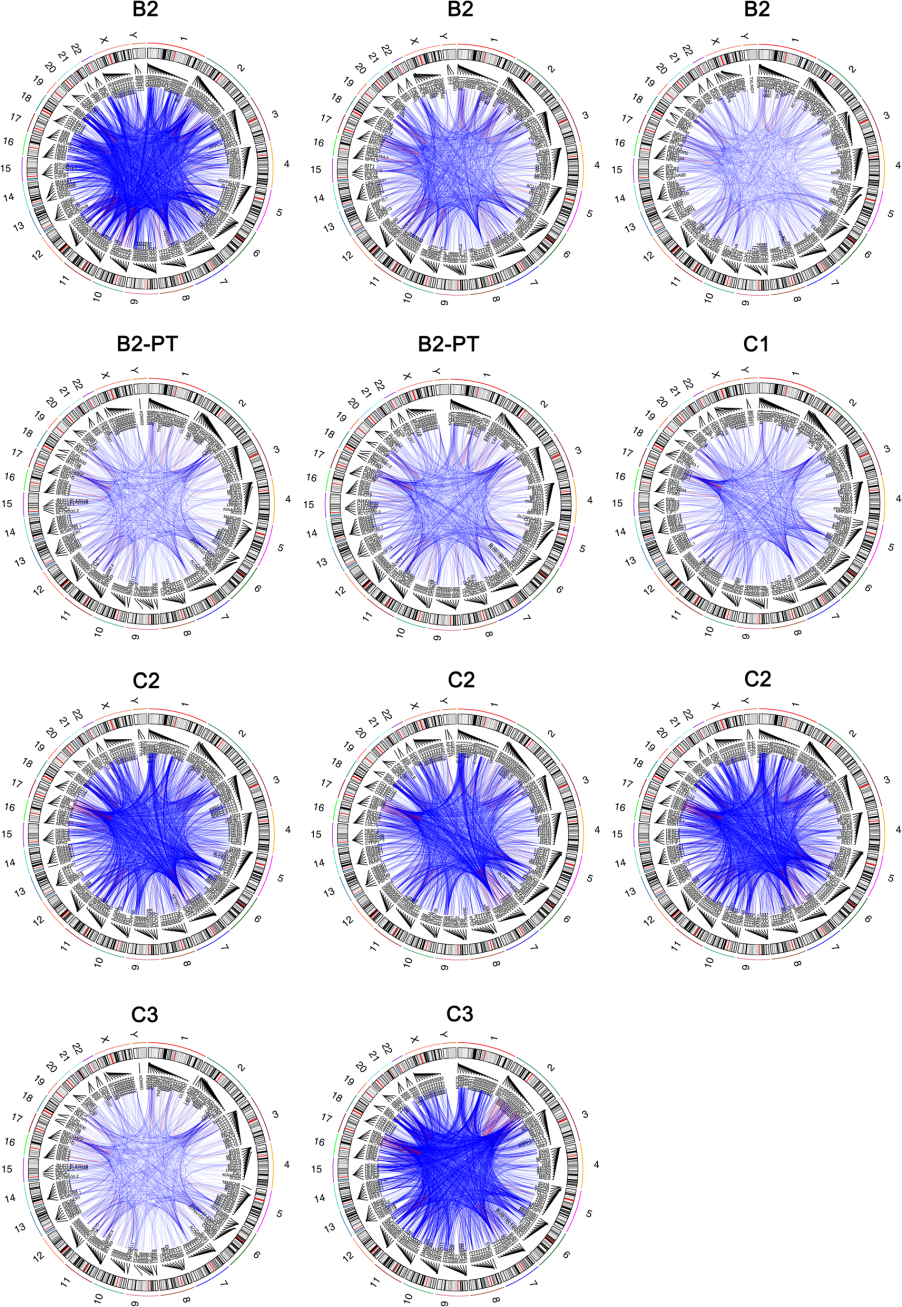
Circos plots of chromosomal rearrangements in all patient tumour core biopsy samples with adequate quality RNA. Fusion genes were identified by carrying out FusionCatcher analysis on RNA-Seq data. Patient B2 and C2 had data from three separate core biopsies, whereas C3 had two and C1 had one core biopsy sample. Patient B2 had two core samples post treatment

**Fig. 4:**
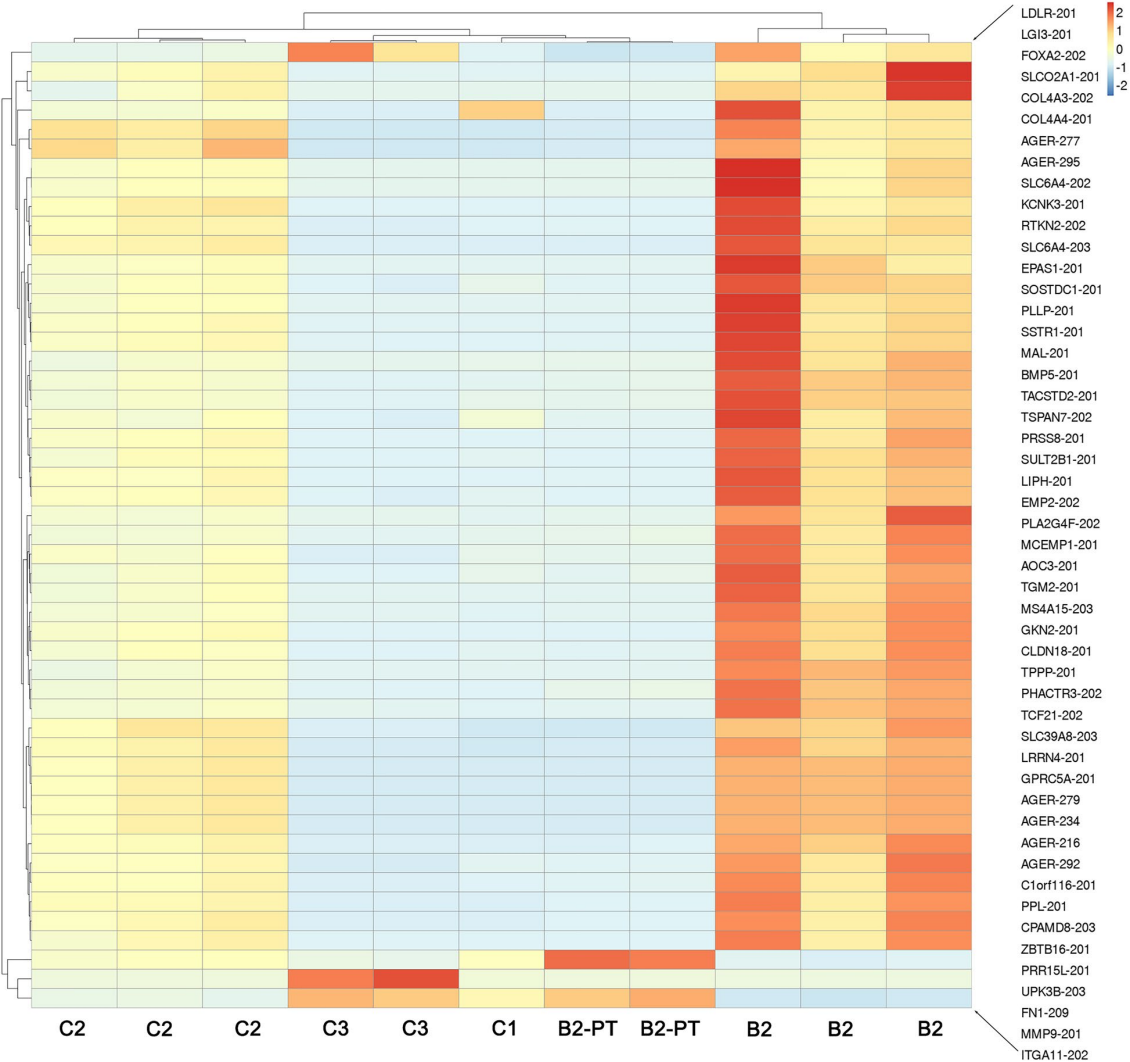
Heatmap of the top 50 most differentially expressed genes amongst the patient biopsy samples. Expression values were calculated using Kallisto. Top and left-hand side dendograms indicate hierarchical clustering of columns (patients) and rows (genes). Note similarity of all three B2 core samples, C2 and C3 samples. Expression changes were identified post treatment in patient B2 (B2-PT)

Tumour samples were collected from 7 patients (Table 4). All 7 patients had tumour samples obtained prior to treatment, but only one sample was obtained post treatment (B2). The reason for this was progressive disease and the reluctance of patients to undergo further biopsies when they would be coming off study. A total of 11 cores from 4 patients (B2, C1–3) obtained from lung, lung VATS procedures and bone biopsies had sufficient RNA quality following processing and shipping that could proceed to RNAseq and germline WES (Table 4). The remaining core biopsy samples were either insufficient or crushed. Analysis for the presence of fusion genes using FusionCatcher showed extensive chromosomal rearrangements consistent with the extreme genomic instability phenotype of osteosarcoma, often referred to as ‘chromothripsis’. Figure 3 illustrates the CIRCOS plots with numerous fusion transcripts (translocations) detected by RNA-Seq in each sample. In patients C1, B2 and C3 the most frequent rearrangements involved chromosomes 2, 5, 6 and 17. It is likely that the heavy involvement of these chromosomal regions reflects the potential generation of numerous driver mutations, as ‘chromothripsis’ and associated amplification of regions on chromosomes 5, 12 and 17 was reported to generate driver events in 37 osteosarcoma genomes [30]. The same RNA-Seq data was analysed for differential gene expression using Kallisto. The heatmap in Fig. 4 shows the top 50 most differentially expressed genes. Despite some heterogeneity in the replicate samples, expression values for the same patient were found to cluster together. Of interest, in the two post-treatment samples from the only patient that had pre and post samples showed changes in gene expression. In patient B2 who received ifosfamide alone in this time period, showed that two functionally related genes, FN1 and ITGA11 had increased expression relative to the pre-treatment samples. FN1, encodes fibronectin, a component of the extracellular matrix and ITGA11, encodes integrin subunit α11, which forms a heterodimer receptor, with β integrin chains, for extracellular matrix proteins. The significance of these observations remain unknown and await further functional analysis. No data was obtained pre and post liposomal MTP-PE in this study, and so the sequencing data was uninformative for the primary biomarker endpoint. Immunostaining with CD14 identified macrophages in all pre-treatment samples, but no comparative analysis was possible with post MTP-PE exposure as there were no paired samples (not shown).

**Table 4:** Summary of all study tissue samples

Patient	Treatment Cycle	Gender	Age	Biopsy location	Number of QC samples for RNAseq^1^
A1	Pre	Male	20	Soft tissue rib - core biopsy	0/2
A3	Pre	Male	23	Lung - core biopsy	0/2
B1	Pre	Male	17	Lung - core biopsy	0/2
B2	Pre	Male	27	Lung - core biopsy	3/3
	Post		27	Lung - core biopsy	2/2
C1	Pre	Male	61	Bone - core biopsy	1/1
C2	Pre	Male	25	Lung – excision biopsy	3/3
C3	Pre	Female	56	Lung & soft tissue – core biopsy	2/2

^a^Samples with RNA integrity (RIN) > 7 using an Agilent Bioanalyzer 2100

### Secondary endpoints

The time to event for progression free and overall survival are shown per patient in Table 3. Progressive disease resulted in death in 2 out of 8 patients during the study period. Two patients withdrew consent, one prior to treatment. There were no grade 5 toxicities, and a single grade 4 toxicity of hypokalaemia in arm B. Serious adverse events included febrile neutropenia, pseudomonas infection, encepthalopathy, hypokalaemia, hypophosphataemia and urinary infection associated with ifosfamide chemotherapy (Arm B and C), Table 5. There was one SAE associated with ifosfamide (febrile neutropenia). Other Grade 3 toxicities included upper respiratory tract infection, flu-like symptoms, fatigue, headache, abdominal discomfort, muscle weakness and hypokalaemia (Supplementary Table 2). A summary of the timing of the worst adverse events for each patient are shown in Fig. 2b.

**Table 5:** Summary of all study serious adverse events

		Arm A (***n***^a^ = 3)				Arm B (***n***^a^ = 2)				Arm C (***n***^a^ = 3)			
		Grade				Grade				Grade			
Category	Event Term	2	3	4	5	2	3	4	5	2	3	4	5
Blood and lymphatic system disorders	Febrile neutropenia										1		
Infections and infestations	Pseudomonas infection										1		
	Urinary tract infection						1						
Metabolism	Hypokalaemia							1					
	Hypophosphataemia						1						
Nervous system disorders	Encephalopathy										1		

^a^Number at risk

**Fig. 5:**
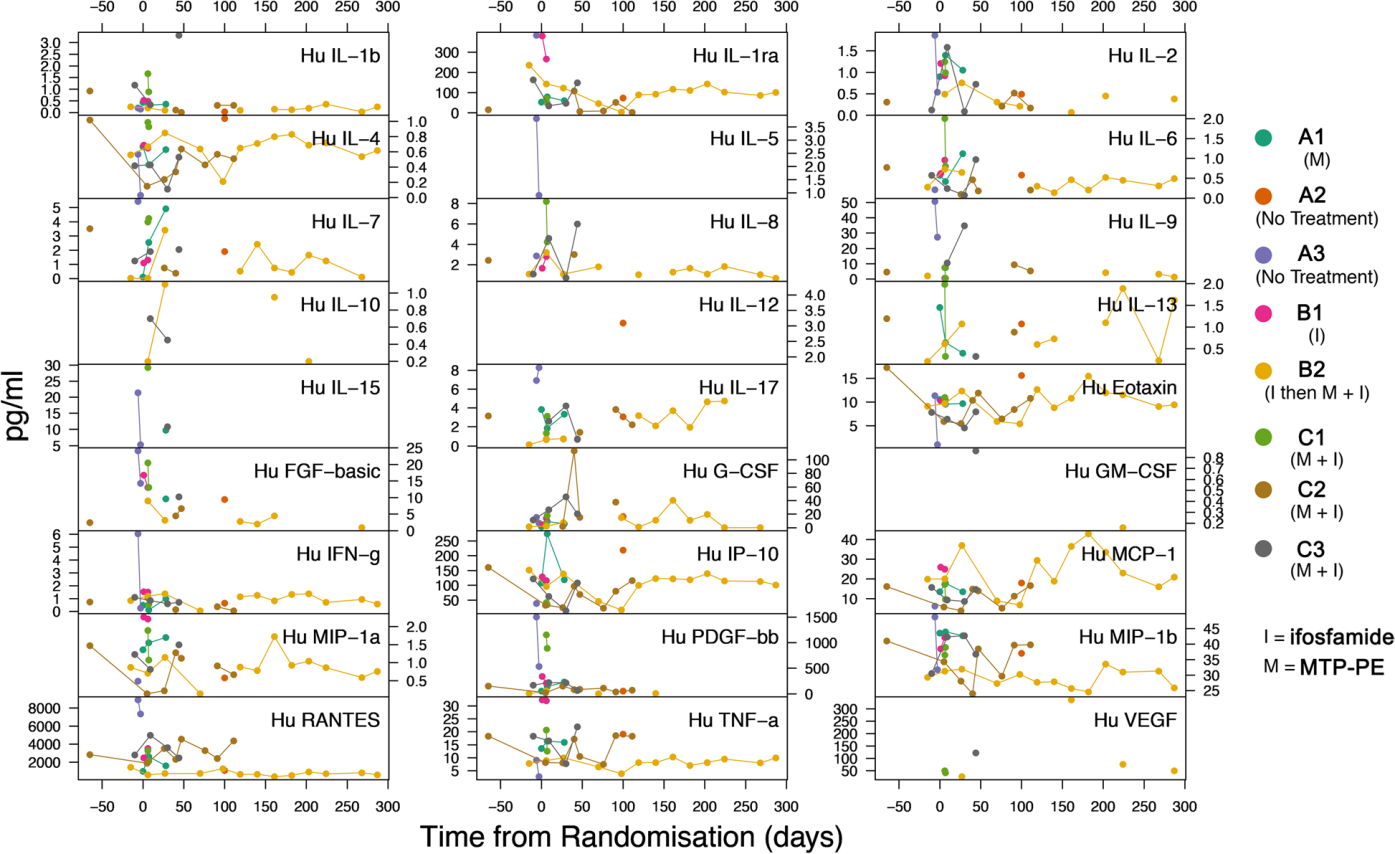
Cytokine activation in peripheral blood samples in different patients (A1 to C3, legend). Results from the Bio-Plex multiplex assay are shown as concentration against time (days) for all collected blood samples for all patients’ available post-randomisation. Samples from specific timepoints are missing either because of failed sample collection or results were below the detection limit of the specific assay

Flu like symptoms were experienced by 3 of the 5 patients receiving liposomal MTP-PE, and all used administered paracetamol as prophylaxis. A total of 34 weekly blood samples for cytokine assays were collected from patients, including all 7 patients at baseline, but only 2 patients (B2 receiving ifosfamide, C2 receiving liposomal MTP-PE plus ifosfamide) to beyond week 6. The results of Luminex cytokine assays in all blood samples are shown in Fig. 5. Macrophage activation as measured by the Bio-Plex multiplex assay can be inferred from increase of cytokines IL-4, G-CSF and MCP-1. IL-4 and MCP1 were not altered upon MTP-PE treatment. However, G-CSF showed an increase, albeit not significant, and probably due to low sample size, suggesting G-CSF mediated macrophage stimulation by MTP-PE. The results overall appear variable and no statistically significant results are obtainable from this data to support analysis of this biological endpoint. The data indicate the feasibility of the analysis and the need for multiple samples from a larger cohort of patients.

## Discussion

The activity of liposomal MTP-PE, consistent with other phase II agents, demonstrates little evidence for response and impact on disease control in metastatic OS [22, 23, 31]. The impact of liposomal MTP-PE in the Phase III (INT-0133) adjuvant study has also been questioned because of a number of study design and outcome questions [32]. The expectation in this study following single agent liposomal MTP-PE in Arm A, was that there may be pathological evidence of response demonstrated by fibrosis and M1 macrophage activation involving the metastatic OS sites. Kleinerman ES et al*,* in 1983 demonstrated that monocytes from osteosarcoma patients could be rendered tumour cytotoxic by both in vitro incubation with MTP-PE and intravenous administration of this agent [12]. Also reported were findings of peripheral fibrosis with neovascularisation and infiltration of the tumour with chronic inflammatory cells that were unlike any observed following chemotherapy or surgery [12]. Viable tumour cells were observed in the centre of the lesion, with necrosis and fibrosis at the periphery. These changes are opposite to that in the treatment naïve tumours, and were thus interpreted as a specific response to MTP-PE. The peripheral fibrosis observed in these tumours was reminiscent of the appearance of pulmonary tuberculosis lesions. Initially, the lesion is walled off and slow necrosis proceeds from the outside so that the lesion is replaced by fibrous tissue. Eradication of tuberculosis by chronic inflammation is a slow process; viable bacilli can persist for months, leading to empirical extension of treatment to six months of therapy. Whether 6 weeks exposure of liposomal MTP-PE would be sufficient could have been assessed if sufficient samples were obtained.

The hypothesis following treatment with a combination of ifosfamide and liposomal MTP-PE, would be evidence of potential synergistic or additive cytotoxicity, and macrophage activation in Arm C compared with Arm B control. In vivo experiments using M1 activated and polarised macrophages with interferon-gamma showed that the combination with liposomal MTP-PE resulted in cell death of human osteosarcoma cell lines [33]. Phase I studies of MTP determined maximum tolerated dose of 4–6 mg/m^2^, with the best biological activity at 0.5–2 mg/m^2^ based on in vitro measures of in vivo stimulation of monocyte tumoricidal activity (MTA) and cytokine release. Whilst the dosage utilised in the study was adequate, the activity of liposomal MTP-PE has not been conclusively demonstrated in metastatic OS, although likely to require combinations with chemotherapy such as doxorubicin, ifosfamide and cisplatin [34–36]. We aimed to determine whether there is a correlation between the tissue specific outcomes and the systemic effects of monocyte/macrophage mediated systemic cytokine release in this study, and remains an important question. The key question remains the spectrum of polarisation of the macrophage population from the M1 (tumour inhibiting) to M2 (tumour promoting). Whilst there is some evidence that M2 can have tumour inhibiting activity specifically in OS lung metastasis, it appears that more detailed evaluation of sub-populations such as CD68−/CD163+ at the single cell level may be a better biological endpoint if the study were to be repeated [37–40]. Indeed, single cell RNAseq has revealed a more complex OS cell type, including co-existing cell types in the tumour microenvironment, opening the prospect of modifiers of the tumour associated M1 and M2 macrophage and immune context that could be combined with liposomal MTP-PE [41, 42]. One consequence of indiscriminate activation of macrophages with liposomal MTP-PE may be the detrimental clinical behaviour of OS, with the risk of selection of different OS somatic clones. Finally, natural killer T cell activity may also have mechanistic impact in OS in this context [43–45].

Integration of clinical trials with tumour biology appears the only way forward to better understand therapeutic targets in these patients, preferably within large sarcoma networks [46]. Here we demonstrate the feasibility of collecting biological samples as the basis of endpoints in a study of metastatic and advanced OS. Baseline samples were adequately collected and of sufficient quality considering the sites of disease in the lung, and as with many studies, post-treatment samples are less frequently achieved dependent on clinical context. It is not uncommon for rare cancer studies to be terminated because of slow recruitment and trial logistics, as these studies often require a large number of sites to recruit a very small number of patients. Moreover, complex therapies requiring chronic administration adds further burden on patients and clinicians, that in effect, bias the types of studies that can be performed in rare cancers. Despite the logistic issues with slow recruitment into this trial, the results of mechanistic studies of liposomal MTP-PE remain important to develop as the agent is used as standard of care in many countries, without a full understanding of the basis of any either tumour promoting or inhibiting effect. The encouraging results of the National Cancer Institute (NCI) and Children’s Oncology Group (COG) phase III clinical study (INT-0133), and the positive results that have been translated into regulatory approval, do hold initial promise for innate immune therapies in osteosarcoma [47]. Further investigation of liposomal MTP-PE in future trials incorporating the experience of this trial in advanced and metastatic osteosarcoma remains warranted.

## Supplementary Information

**Additional file 1.**

**Additional file 2.**

## Data Availability

The anonymised genomics data (including all of the raw Fastq files) will be deposited in the EMBL-EBI European Nucleotide Archive (https://www.ebi.ac.uk/ena/browser/home) under accession PRJEB53234 where it will remain in the public domain. Anonymised data for the study is stored under current data protection legislation in Oxford University servers may be available on formal request.
