# Supplementary material for: Outcomes from a mechanistic biomarker multi-arm and randomised study of liposomal MTP-PE (Mifamurtide) in metastatic and/or recurrent osteosarcoma (EuroSarc-Memos trial)
Source: BMC Cancer. 2022 Jun 8;22:629. doi: 10.1186/s12885-022-09697-9 (PMC9175372; doi:10.1186/s12885-022-09697-9)
Supplement: Supplementary file 1 — Additional file 1. [file 12885_2022_9697_MOESM1_ESM.docx]

**Supplementary Tables**

**Supplementary Table 1. CONSORT Abstract summary**

**Outcomes from a mechanistic biomarker multi-arm and randomised study of liposomal MTP-PE (Mifamurtide) in metastatic and/or recurrent osteosarcoma (EuroSarc-Memos trial)**

| Authors | Prof Andrew (Bass)im Hassan; [bass.hassan@path.ox.ac.uk](mailto:bass.hassan@path.ox.ac.uk)  Professor of Medical Oncology, Oxford Molecular Pathology Institute and Oxford Biomedical Research Centre, Sir William Dunn School of Pathology, University of Oxford, OX1 3RE, UK | | | |
| --- | --- | --- | --- | --- |
| Trial design | Interventional Phase 2  Allocation: Randomised Intervention Model: Parallel Assignment Masking: None (Open Label) Primary Purpose: Treatment | | | |
| Methods: |  | | | |
| Participants | Setting of bone sarcoma clinical trials units based in cancer hospitals in Europe.  Inclusion Criteria:   1. Relapsed osteosarcoma (first, second, third or any relapse, patient has recovered from chemotherapy and any other investigational drug/agent treatment, radiotherapy or surgical procedure). 2. Histological confirmed diagnosis of osteosarcoma at original presentation. 3. Tumour at biopsy accessible or resectable site. 4. Progressive disease documented by imaging within 3 months of entry into the trial. 5. At least one measurable lesion on CT scan (RECIST) performed in past 21 days prior to trial entry. 6. Male or female, age ≥ 16 years to 65 (or ≥18 based on institutional practice for Teenage and Young Adult Cancer patients). 7. Life expectancy of at least 3 months. 8. WHO performance score of 0 - 2. 9. The patient is willing and able to comply with the protocol and scheduled follow-up visits and examinations. 10. Written (signed and dated) informed consent. 11. Cardiac shortening fraction ≥ 28% or ejection fraction ≥ 45% 12. Renal function is adequate for ifosfamide treatment (GFR as per table below, other renal function screening tests as per local practice) 13. Haematological and biochemical indices within the ranges shown below:   Lab Test Value required   - Haemoglobin (Hb) ≥ 9 g/dL (Previous transfusion is allowed) - Absolute neutrophil count (ANC) >=1.0 x 10*9/L without growth factor support - Platelet count > 80.x 10*9/L (Previous transfusion is allowed) - Total bilirubin <1.5 times the upper limit of normal (ULN) for age (except for Gilbert's syndrome patients) - Serum alanine aminotransferase (ALT) and/or Aspartate aminotransferase (AST) <2.5 × ULN for age, <2.5 × ULN for age - Serum creatinine Normal range for age - Glomerular filtration rate (GFR) (calculated as 51Cr-EDTA/99mTc-DTPA clearance) >40ml/min if deemed resectable (for Arm A), >60ml/min if not deemed resectable (for Arm B or C)   Exclusion Criteria:   1. Pregnant or breast-feeding woman. Men or women of childbearing potential unless effective methods of contraception are used during study treatment and for at least 7 days after the last mifamurtide dose (see section 5.1 Informed consent - Contraceptive/ Pregnancy counselling). 2. Previous treatment with mifamurtide or a mifamurtide-like drug* in a clinical trial setting for the treatment of metastatic and/or recurrent osteosarcoma in the six months prior to registration. 3. Contraindications to lung biopsies. 4. Hypersensitivity to ifosfamide or any component of the formulation. 5. Previously diagnosed brain metastases. 6. Significant active cardiac disease including: uncontrolled high blood pressure (no greater than 2 standard deviations above the mean for age for systolic blood pressure (SBP) and diastolic blood pressure (DBP), unstable angina, congestive heart failure, myocardial infarction within the previous 6 months, or serious cardiac arrhythmias and with a history of pericarditis and myocarditis 7. Treatment with any other investigational agent, or participation in another interventional clinical trial within 21 days prior to enrolment. 8. Major surgery within 21 days prior to first study biopsy 9. Currently taking high-dose non-steroidal anti-inflammatory drugs (NSAIDs) or corticosteroid treatment 10. Concurrent use of ciclosporin or other calcineurin inhibitors. 11. Any psychological, social or medical condition, physical examination finding or a laboratory abnormality that the Investigator considers would make the patient a poor trial candidate or could interfere with protocol compliance or the interpretation of trial results. 12. Any other active malignancy, with the exception of adequately treated cone-biopsied in situ carcinoma of the cervix uteri and non-melanoma skin lesions. 13. Patients who are known to be serologically positive for Hepatitis B, Hepatitis C or HIV.     - mifamurtide-like drugs include GCSF, GMCSF, interferon and other macrophage activating molecules. | | | |
| Interventions | **Experimental: Group A. Resectable:** Mifamurtide only  Treatment Weeks 1-6 (post 1st biopsy/resection):  Mifamurtide 2mg/m2, IV infusion, twice/week, with each infusion given at least 3 days apart, for 6 weeks.  Treatment Weeks 7-12 (post 2nd biopsy/resection):  Mifamurtide 2mg/m2, IV infusion, twice/week, with each infusion given at least 3 days apart, for 6 weeks.  Treatment Weeks 13-36:  Mifamurtide 2mg/m2, IV infusion, once/week.  Intervention: Drug: Mifamurtide  **Experimental: Group B. Unresectable Control**: Ifosfamide (Followed by Mifamurtide)  Treatment Weeks 1-6: Day 1 of 21: Ifosfamide 12-15g/m2 IV infused over 4-5 days as per local practice. Repeated every 21 days for 2 cycles (3 weeks=1 cycle).  Treatment Weeks 7-12 (post 2nd biopsy/resection): Day 1 of 21: Ifosfamide 12-15g/m2 IV infused over 4-5 days once every 21 days for two cycles (3 weeks=1 cycle). Ifosfamide administered as per local practice, including concurrent dosing with mesna. Plus mifamurtide 2mg/m2, IV infusion, twice/week. Ifosfamide infusion started 24 hours prior to mifamurtide. Mifamurtide given on day 2 and either day 5 or day 6.  Treatment Weeks 13-18: Mifamurtide 2mg/m2, IV infusion, twice/week. Treatment Weeks 19-42: Mifamurtide 2mg/m2, IV infusion, once/week.  Interventions:   - - Drug: Mifamurtide   - Drug: Ifosfamide   **Experimental: C. Unresectable Experimental**: Ifosfamide + Mifamurtide  Treatment Weeks 1-6:  Day 1 of 21: Ifosfamide 12-15g/m2 IV infusion over 4-5 days once every 21 days for two cycles (3 weeks=1 cycle).  Plus Mifamurtide 2mg/m2, IV infusion, twice per week, each given at least 3 days apart, for 6 weeks.  Ifosfamide infusion started 24 hours prior to mifamurtide. Mifamurtide given on day 2 and either day 5 or day 6.  Treatment Weeks 7-12 (post 2nd biopsy/resection):  Day 1 of 21: Ifosfamide 12-15g/m2 IV infusion over 4-5 days once every 21 days for two cycles (3 weeks = 1 cycle).  Plus Mifamurtide 2mg/m2, IV infusion, twice per week, given at least 3 days apart, for 6 weeks. Ifosfamide infusion started 24 hours prior to mifamurtide. Mifamurtide given on day 2 and either day 5 or day 6.  Treatment Weeks 13-36: Mifamurtide 2mg/m2, IV infusion, once/week.  Interventions:   - - Drug: Mifamurtide   - Drug: Ifosfamide | | | |
| Objective | The objective being tested was to identify markers of response to MTP-PE (mifamurtide) by evaluation of radiological response and biological markers of immune response activation in tumour samples, taken before and after six weeks of treatment. The pharmacodynamic readouts were to be compared with radiological (CT) scan response using standard RECIST criteria. A patient was defined as a responder if at least one of these two endpoints was met and a non-responder otherwise, (on an intention to treat basis). | | | |
| Outcome | 1. Biological Response Data Based on Pharmacodynamic Endpoints on Tumour Biopsy Material [ Time Frame: Change from Baseline to after 6 weeks of treatment ]   Biological response data based on pharmacodynamic endpoints on tumour biopsy material including macrophage infiltration and innate immune activation.   1. Radiological Response Defined as Complete or Partial Response and Assessed Using RECIST Criteria [ Time Frame: Change from Baseline to after 6 weeks of treatment ]   Per Response Evaluation Criteria In Solid Tumors Criteria (RECIST v1.1) assessed by CT or MRI:  Complete Response (CR): Disappearance of all target and non-target lesions Partial Response (PR): >=30% decrease in the sum of the longest diameter of target lesions, AND no evidence of progression in non-target lesions, AND no new lesions Stable Disease (SD): sum of longest diameter of target lesions between PR and PD values, AND no evidence of progression in non-target lesions, AND no new lesions Progressive Disease (PD): >20% increase in the sum of the longest diameter of target lesions, OR evidence of progression in non-target lesions, OR evidence of new lesions | | | |
| Randomisation | Computer generated block randomisation to arm B or C by Sponsor clinical trial office. | | | |
| Blinding (masking) | Open label unblinded | | | |
| Results: |  | | | |
| Numbers randomised | Group B: 2  Group C: 3 | | | |
| Recruitment | Trial First recruitment 15/05/2015  Trial Completion 04/11/2017  18 assessed for eligibility  10 patients excluded (4 did not meet inclusion/exclusion criteria, 6 declined to participate). | | | |
| Numbers analysed | Number | Group A resectable  (M) | Group B unresectable  (I) | Group C unresectable  (I+M) |
|  | Started | 3 | 2 | 3 |
|  | Scan  (6 weeks) | 2 | 1 | 2 |
|  | Completed | 0 | 0 | 0 |
|  | Not Completed | 3 | 2 | 3 |
|  | Lack of efficacy | 2 | 1 | 2 |
|  | G3+Adverse event | 0 | 3 | 3 |
|  | Consent withdrawn | 1 | - | 1 |
| Outcome | End points | Group A resectable (M) | Group B unresectable (I) | Group C unresectable  (I+M) |
|  | Number | 3 | 2 | 3 |
|  | SD | 0 | 1 | 1 |
|  | PD | 2 | 0 | 1 |
|  | Did not reach endpoint | 1 | 1 | 1 |
|  | Progressed before endpoint | 2 | 0 | 0 |
|  | Macrophage count | 0 | 0 | 0 |
| Harms | Serious Adverse Events | Group A resectable (M) | Group B unresectable (I) | Group C unresectable  (I+M) |
|  | Number exposed | 3 | 2 | 3 |
|  | Death from adverse events | 0/0 | 0/0 | 0/2 |
|  | CTCAE Grade 3+ | 0 | 3 | 3 |
|  |  |  | Hypo-phosphataemia, hypokalemia, UTI | Encephalopathy, febrile neutropenia, pseudomonas infection |
| Conclusions | The number of rare patients that could be recruited in the funding period did not meet recruitment targets and required the trial to stop. This was despite opening additional sites. The samples could be collected and preliminary biomarker analysis can be performed if a future trial was undertaken. | | | |
| Trial registration | NCT02441309 12/05/2015, ISRCTN46249783, EudraCT 2012-000615-84, EuroSarc-MEMOS | | | |
| Funding | EuroSarc EU 7th Framework Programme (Grant agreement number 278742, Principal Investigator, Prof JY Blay, Universitè Lyon 1 Claude Bernard, Lyon, France) | | | |

**Supplementary Table 2. Summary of all adverse events**

|  |  | **Arm A**  **(n = 3)** | | | | **Arm B**  **(n = 2)** | | | | **Arm C**  **(n = 3)** | | | |
| --- | --- | --- | --- | --- | --- | --- | --- | --- | --- | --- | --- | --- | --- |
| **Category** | **Event Term** | **1** | **2** | **3** | **4** | **1** | **2** | **3** | **4** | **1** | **2** | **3** | **4** |
| Blood and lymphatic system disorders | Febrile neutropenia | 1 |  |  |  | 1 | 1 |  |  | 1 | 1 | 1 |  |
| Gastrointestinal disorders | Abdominal discomfort |  |  |  |  |  |  |  |  | 1 | 1 |  |  |
|  | Abdominal pain |  |  |  |  | 1 |  |  |  |  |  |  |  |
|  | Haemorrhoids |  |  |  |  | 1 |  |  |  |  |  |  |  |
|  | Nausea |  |  |  |  |  |  |  |  | 1 | 1 |  |  |
|  | Vomiting |  |  |  |  | 1 |  |  |  |  |  |  |  |
| General disorders and administration site conditions | Chest pain |  |  |  |  | 1 | 1 |  |  |  |  |  |  |
|  | Fatigue |  |  |  |  | 1 |  |  |  |  |  |  |  |
|  | Fever |  |  |  |  | 1 | 1 |  |  | 1 | 1 |  |  |
|  | Flu like symptoms | 1 |  |  |  |  |  |  |  |  |  |  |  |
|  | Shivering |  |  |  |  | 1 |  |  |  |  |  |  |  |
| Infections and infestations | Central line infection |  |  |  |  |  |  |  |  | 1 |  |  |  |
|  | Infected toe |  |  |  |  | 1 | 1 |  |  |  |  |  |  |
|  | Pseudomonas infection |  |  |  |  | 1 | 1 |  |  |  |  |  |  |
|  | Upper respiratory tract infection |  |  |  |  |  |  |  |  | 1 | 1 |  |  |
|  | Urinary tract infection |  |  |  |  | 1 |  |  |  | 1 | 1 |  |  |
| Metabolism and nutrition disorders | Hypokalaemia |  |  |  |  | 1 | 1 | 1 |  |  |  |  |  |
|  | Hypophosphataemia |  |  |  |  | 1 | 1 | 1 | 1 |  |  |  |  |
| Musculoskeletal and connective tissue disorders | Muscle weakness |  |  |  |  | 1 | 1 |  |  |  |  |  |  |
| Nervous system disorders | Encephalopathy |  |  |  |  | 1 | 1 |  |  | 11 | 1 | 1 |  |
|  | Headache |  |  |  |  |  |  |  |  | 1 | 1 |  |  |
|  | Headaches | 1 |  |  |  | 1 | 1 |  |  |  |  |  |  |
|  | Taste altered |  |  |  |  |  |  |  |  | 1 | 1 |  |  |
| Respiratory, thoracic and mediastinal disorders | Pneumothorax |  |  |  |  | 1 |  |  |  |  |  |  |  |
|  | Shortness of breath |  |  |  |  |  |  |  |  | 1 | 1 |  |  |
| Skin and subcutaneous tissue disorders | Alopecia |  |  |  |  | 1 |  |  |  |  |  |  |  |
| Vascular disorders | Hypotension |  |  |  |  | 1 |  |  |  | 1 | 1 |  |  |

Table reports worst grade per patient and are cumulative. For example, in arm B at baseline one patient had at least a grade 2. Every patient who had a grade 2 will also be included in the column for at least a grade 1. We can deduce that one patient had at least one adverse event with grade 2 and one patient had no adverse events at baseline. No patients had a grade 3 at baseline.
