# Supplementary material for: Outcomes from a mechanistic biomarker multi-arm and randomised study of liposomal MTP-PE (Mifamurtide) in metastatic and/or recurrent osteosarcoma (EuroSarc-Memos trial)
Source: BMC Cancer. 2022 Jun 8;22:629. doi: 10.1186/s12885-022-09697-9 (PMC9175372; doi:10.1186/s12885-022-09697-9)

Supplementary Tables and Figures

Supplementary Table 1. Summary of adverse events

		Arm A (n = 3)				Arm B (n = 2)				Arm C (n = 3)			
**Category**	**Event Term**	**1**	**2**	**3**	**4**	**1**	**2**	**3**	**4**	**1**	**2**	**3**	**4**
Blood and lymphatic system disorders	Febrile neutropenia	1				2				3			
Gastrointestinal disorders	Abdominal discomfort									1	1	1	
	Abdominal pain					1							
	Haemorrhoids					1							
	Nausea									1	1		
	Vomiting					1							
General disorders and administration site conditions	Chest pain					2							
	Fatigue					1							
	Fever					1	1			1	1		
	Flu like symptoms	1											
	Shivering					1							
Infections and infestations	Central line infection									1			
	Infected toe					1	1						
	Pseudomonas infection					1	1						
	Upper respiratory tract infection									1	1	1	
	Urinary tract infection					1							
Metabolism and nutrition disorders	Hypokalaemia					1	1	1					
	Hypophosphataemia					1	1	1	1				
Musculoskeletal and connective tissue disorders	Muscle weakness					1	1	1					
Nervous system disorders	Encephalopathy					1	1						
	Headache									1	1	1	
	Headaches	1				1	1						
	Taste altered									1	1		
Respiratory, thoracic and mediastinal disorders	Pneumothorax					1							
	Shortness of breath									1	1		
Skin and subcutaneous tissue disorders	Alopecia					1							
Vascular disorders	Hypotension					1				1	1		

Table reports worst grade per patient and are cumulative. For example, in arm B at baseline one patient had at least a grade 2. Every patient who had a grade 2 will also be included in the column for at least a grade 1. We can deduce that one patient had at least one adverse event with grade 2 and one patient had no adverse events at baseline. No patients had a grade 3 at baseline.

Supplementary Figure 1. Summary of all adverse events per patient

The vertical black lines denote the time of treatment or study withdrawl per patient. Two adverse free patients in Arm 1 are represented by the empty rows.

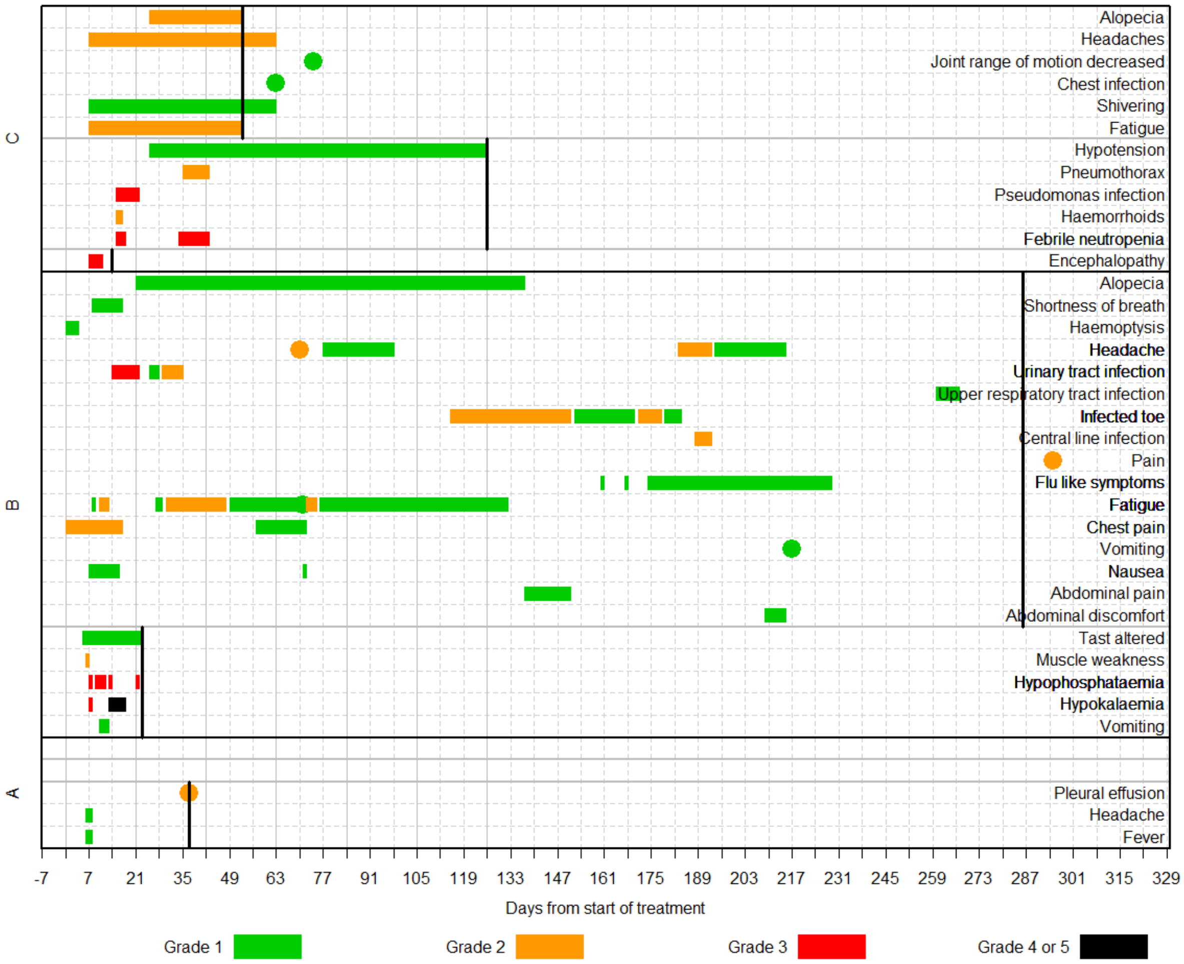

Supplement: Supplementary file 2 — Additional file 2. [file 12885_2022_9697_MOESM2_ESM.pdf]
